# Incentives for Delay-Constrained Data Query and Feedback in Mobile Opportunistic Crowdsensing

**DOI:** 10.3390/s16071138

**Published:** 2016-07-21

**Authors:** Yang Liu, Fan Li, Yu Wang

**Affiliations:** 1 School of Automation, Beijing Institute of Technology, 5 South Zhongguancun Street, Haidian District, Beijing 100081, China; 2 School of Computer Science and Technology, Beijing Institute of Technology, 5 South Zhongguancun Street, Haidian District, Beijing 100081, China; fli@bit.edu.cn; 3 College of Computing and Informatics, University of North Carolina at Charlotte, 9201 University City Blvd., Charlotte, NC 28223-0001, USA; yu.wang@uncc.edu

**Keywords:** mobile opportunistic crowdsensing, incentive mechanism, data query, two-person cooperative game, optimal stopping theory

## Abstract

In this paper, we propose effective data collection schemes that stimulate cooperation between selfish users in mobile opportunistic crowdsensing. A query issuer generates a query and requests replies within a given delay budget. When a data provider receives the query for the first time from an intermediate user, the former replies to it and authorizes the latter as the owner of the reply. Different data providers can reply to the same query. When a user that owns a reply meets the query issuer that generates the query, it requests the query issuer to pay credits. The query issuer pays credits and provides feedback to the data provider, which gives the reply. When a user that carries a feedback meets the data provider, the data provider pays credits to the user in order to adjust its claimed expertise. Queries, replies and feedbacks can be traded between mobile users. We propose an effective mechanism to define rewards for queries, replies and feedbacks. We formulate the bargain process as a two-person cooperative game, whose solution is found by using the Nash theorem. To improve the credit circulation, we design an online auction process, in which the wealthy user can buy replies and feedbacks from the starving one using credits. We have carried out extensive simulations based on real-world traces to evaluate the proposed schemes.

## 1. Introduction

With the improvement in hardware manufacturing, CPU architectures, radio communication techniques and software design, the price of smartphones can be accepted by the majority of people, resulting in large availability. Due to powerful computation, communication capabilities and various functional built-in sensors, smartphones enable accurate trace world-related information and activities of citizens by taking advantage of people willing to collaborate toward continuous data collection, called crowdsensing [1]. The rapid development and proliferation of sensor-equipped smartphones are making mobile crowdsensing an effective way to enable more and more applications, ranging from L3 [2] for traffic monitoring, iCal [3] for noise monitoring, vCity Map [4,5] for smart cities [6], for air pollution monitoring, LiFS [7] for indoor localization and [8] for urban WiFi characterization.

We consider mobile opportunistic crowdsensing formed by mobile users who share similar interests and connect with one another by exploiting Bluetooth and/or WiFi connections of their mobile phones or portable tablets. Mobile opportunistic crowdsensing is often created for a local community where the participants have frequent interactions, e.g., people living in an urban neighborhood, students studying in a college or tourists visiting an archaeological site. Its size varies from a large group (for instance, all students in a university) to a small cluster (such as members of a school band). It may serve a community over a long span of years or be temporary, lasting for as short as a few hours only. As discussed in the context of delay-/disruption-tolerant networks (DTNs) [9,10,11], sporadically-connected sensor networks [12], vehicular networks [13], peer-to-peer mobile social networks [14,15,16], and 5G networks [17,18,19], mobile opportunistic crowdsensing is characterized by intermittent and nondeterministic connectivity, often due to interruptible wireless links, sparse network deployment and/or nodal mobility.

### 1.1. Incentives and Selfishness

Mobile users in mobile opportunistic crowdsensing can be either cooperative or selfish. Each cooperative user carries data packets for others voluntarily. However, if a user is selfish, it is often reluctant to consume its energy, storage and bandwidth resources for other users, resulting in poor performance. The more users help others deliver data packets, the better the network performance. Therefore, it is imperative to propose an incentive scheme to stimulate user cooperation.

In this work, we consider users selfish and rational. The data query is a “push-and-pull” model, where a query issuer intends to query data. At the same time, each data provider has expertise for each category to reply to queries. In practice, however, it is nontrivial to properly define the expertise, because a mobile user hardly knows precisely his or her probability to answer queries in each category. He or she may initially claim his or her expertise based on the mobile user’s social roles. However, such initially claimed expertise is often inaccurate. Therefore, data providers intend to require feedbacks from query issuers. Both query issuers and data providers pay for the delivery service. Other users participate in the query, reply and feedback delivery only if they are beneficiaries. This is in a contrast to other incentive models in the literature, where either sources intend to “push” data as payers or receivers intend to “pull” data and, thus, are deemed as payers [20,21,22]. In this paper, we assume that neither do all users consume their resources to help, nor to maliciously attack others. We also assume strong authentication that provides auditability for the verification of the identities of users and prevents forging identification to obtain a free forwarding service or more rewards from others.

**Figure 1:**
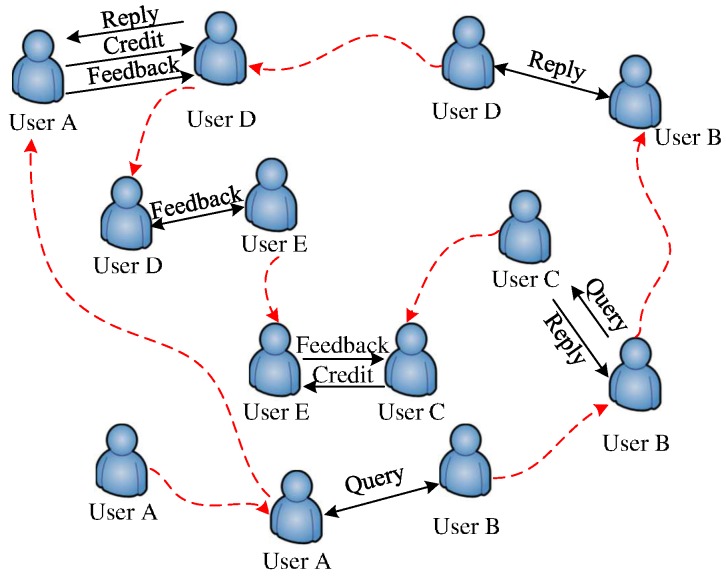
An example of data query and feedback in a small community where the red dotted curved arrow indicates the movement of a user, and the black solid straight line arrow depicts communication.

An example is illustrated in Figure 1, where User *A* wonders “who can get the ticket for the Superbowl final” and would like to query the data in his or her community. Therefore, User *A* generates a query for sports. Obviously, User *A* needs to get the ticket before the final; therefore, the delay budget for the query is from the date User *A* generates the query to the final date. When User *A* meets User *B*, they trade the query by a trading process. The reason that User *B* is eager to obtain the query is that User *B* can meet User *C* frequently, who has expertise for sports news. Therefore, when User *B* meets User *C*, User *B* sends the query to User *C* and gets the reply from it. After the retrieval of the reply, User *B* has two ways to get paid from User *A*. On the one hand, User *B* can cash in the reply when User *B* meets User *A* directly. However, it will be infeasible if User *B* has a small contact probability with User *A*. On the other hand, User *B* may trade the reply with another user (e.g., User *D*). Such a trading process should benefit both users. User *D* gets paid from User *A* when they meet; at the same time, User *A* sends a feedback to User *D*. When User *D* meets User *E*, the feedback will be exchanged via the same trading process, if the process benefits both users. Finally, User *C* cashes in the feedback to User *E* when User *C* meets User *E*. Note that queries, replies and feedbacks can be traded when two users meet. However, it is difficult to determine which data packet should be traded, since selfish users aim to maximize their own benefits only. Therefore, when a user wants to require a data packet from another user, he or she needs to trade one data packet of his or her own. The two-user trading process can be formulated as a two-person cooperative game; given the selfishness of the two users, the binding agreement must do good for both users.

### 1.2. Contribution of this Work

We propose incentive schemes for data query in mobile opportunistic crowdsensing. A query issuer generates a query and requests replies within a given delay budget. When a data provider receives the query for the first time from an intermediate user, the former replies to it and authorizes the latter as the owner of the reply. Multiple copies of a query can be created and replied to by different data providers. When a user that owns a reply meets the query issuer that generates the query, it requests the query issuer to pay credits. Note that a query issuer only pays replies issued by himself or herself. The query issuer pays credits and provides a feedback of the reply to the data provider. Each user has an expertise for each category. In practice, however, it is nontrivial to properly define the expertise, because a mobile user hardly knows precisely his or her probability to answer queries in each category. He or she may initially claim his or her expertise based on the mobile user’s social roles (e.g., professions), interests and available resources. However, such initially claimed expertise is often inaccurate. Therefore, after initialization, the expertise should be updated according to the feedbacks from the query issuers. When a user that carries the feedback meets the data provider that relates to the feedback, the data provider pays credits to the user in order to adjust his or her claimed expertise. Queries, replies and feedback can be traded between mobile users. Multiple replies of a query can be obtained by different data providers. Only the first copy of the reply can be payed credits. On the other hand, the first copy of the feedback can be cashed back. Therefore, the key point is how to effectively track the potential value of queries, replies or feedbacks and how to have them get payed as quickly as possible in such an intermittent connectivity setting. We propose an effective mechanism to define rewards for queries, replies and feedbacks. We formulate the bargain process as a two-person cooperative game, whose solution is found by using the Nash theorem. We carry out extensive simulations to evaluate the proposed schemes under real-world mobility traces.

The rest of the paper is organized as follows: Section 2 discusses related work. Section 3 introduces our proposed incentive scheme. Section 4 develops an online auction algorithm based on the optimal stopping strategy. Section 5 presents simulations under real-world mobility traces. Finally, Section 6 concludes the paper.

## 2. Related Work

Dealing with selfish users has been extensively studied in the context of mobile Ad Hoc networks [23,24,25,26]. The work in [23] proposes a reputation-based approach, where the reputation of each node reflects its degree of cooperation. Nodes update their reputation by forwarding packets for other nodes and select a routing path based on nodal reputation. The work in [24,25,26] develop credit-based approaches, where a node earns credits by delivering packets for others and uses such credits to obtain the data delivery service from other nodes in the network. However, these incentive approaches are not directly applicable in mobile opportunistic crowdsensing. The intermittent connectivity in mobile opportunistic crowdsensing makes it impractical for a node to build up the reputation of its neighbors as required in the reputation-based approaches or to estimate the number of intermediate nodes that would be involved in packet forwarding as required in the credit-based schemes.

Several incentive works are developed for mobile crowdsensing. For example, the work in [27] takes advantage of the pervasive smartphones to collect data. They consider two system models: the platform-centric model where the platform provides a reward shared by participating users and the user-centric model where users have more control over the payment they will receive. For the platform-centric model, they design an incentive mechanism using a Stackelberg game, where the platform is the leader, while the users are the followers.

The work in [28] proposes an online incentive mechanism design for crowdsensing applications with smartphones, where the platform does not have to synchronize large amounts of users simultaneously while distributing tasks. The work in [29] investigates two-sided online interactions among service users and service providers in mobile crowdsourcing. They model such interactions as online double auctions, explicitly taking the dynamic nature of both users and providers into account, and propose a general framework for the design of truthful online double auctions for dynamic mobile crowdsourcing. Clearly, the above scenarios are different from this work, where data query in mobile opportunistic crowdsensing results in a distinctive communication paradigm characterized by intermittent link connectivity, autonomous computing storage and unknown or inaccurate expertise, making data query in mobile opportunistic crowdsensing a very unique, interesting and challenging problem. Only a handful of works have considered data query in opportunistic network settings. The work in [30] employs the epidemic approach for query dissemination in the network, and the replies are routed back based on the traces while traveling between query issuers and data providers. The work in [31] proposes to query geo-location-based information, where each node moves according to a given schedule and adopts a semi-Markov model to predict nodal meeting events, in order to identify a proper relay to carry the query to the target location and bring the interested information back to the source. However, neither of them considers incentives for the data query in mobile opportunistic crowdsensing.

## 3. Proposed Incentive Scheme for Delay-Constrained Data Query and Feedback

In this section, we first introduce some preliminaries, then propose our incentive scheme. To make the definitions clearer, we list them in Table 1.

### 3.1. Preliminaries

#### 3.1.1. Deliveries

Each query is associated with a number of deliveries, which indicates the number of the intended data providers that can reply to the query. Let $\gamma_{q}$ denote the deliveries of Query *q*, which can be learned by a counting algorithm [32]. Each feedback maintains a delivery number, which is an indicator of the current estimation of the copies of the feedback in the network. Let $\gamma_{f}$ denote the deliveries of Feedback *f*. $\gamma_{f}$ can be obtained by a split-based approach [33].

#### 3.1.2. Appraisal

Each a copy of a query is associated with an appraisal, which indicates the amount of credits the query issuer is willing to pay to each intended data provider that replies to the query. Let $\lambda_{q}$ denote the appraisal of Query *q*. Similarly, each feedback maintains an appraisal that indicates the number of credits the data provider is willing to pay to the user that delivers the feedback. Let $\lambda_{f}$ denote the appraisal of Feedback *f*.

#### 3.1.3. Delay-Constrained Category Contact Probability

The delay-constrained category contact probability (DCCP) indicates the probability that User *i* delivers queries of Category *c* to data providers within a given delay budget directly or indirectly. Its value intrinsically depends on the aggregated direct and indirect delay-constrained contact likelihood with data providers. The former, i.e., the direct delay-constrained category contact probability of User *i* in Category *c*, represents the probability that User *i* directly meets a data provider that can reply to the queries in Category *c* within a given delay budget. The latter is the indirect delay-constrained category contact probability, which indicates the probability that User *i* delivers the queries to the data provider via other users indirectly within a given delay budget. In this research, we adopt the exponentially-weighted moving average (EWMA), which is an effective scheme for online estimation to maintain and update the delay-constrained category contact probability.

The delay-constrained category contact probability is intrinsically the cumulative distribution function of delivery delay [34], which is ideal to support QoS data delivery, but impractical to maintain in continuous time under an arbitrary delay distribution. Thus, we adopt discrete time slots to construct approximate delay distributions, where a slot is Δ minutes. The delay distribution of a direct link between User *i* and a data provider in Category *c* can be represented by a vector $\left[P_{ic}^{1},P_{ic}^{2},...,P_{ic}^{K}\right]$, where $P_{ic}^{k}$ is the probability that their inter-meeting time is greater than (k-1)Δ and less than kΔ. Such delay distributions can be built via a trivial online learning algorithm according to historical inter-meeting times. Let $P_{i}^{c}\left(\delta \right)$ denote the direct delay-constrained contact probability of User *i* with data providers in Category *c* with remaining Delay Budget *δ*. When User *i* meets User *j* that is not a data provider in Category *c*, User *i* maintains the delay distribution between User *i* and User *j*, i.e., $\left[P_{ij}^{1},P_{ij}^{2},...,P_{ij}^{K}\right]$, User *j* maintains the delay distribution between User *j* and the data provider in Category *c*, i.e., $\left[P_{jc}^{1},P_{jc}^{2},...,P_{jc}^{K}\right]$; thus, the indirect delay distribution from User *i* to Data Provider *c* via User *j* can be calculated as the convolution of $\left[P_{ij}^{1},P_{ij}^{2},...,P_{ij}^{K}\right]$ and $\left[P_{jc}^{1},P_{jc}^{2},...,P_{jc}^{K}\right]$. Let ${\bar{P}}_{i}^{c}\left(\delta \right)$ denote the indirect delay-constrained contact probability of User *i* with data providers in Category *c* with remaining Delay Budget *δ*. As shown in [35], two-hop relaying achieves the most performance gains. Therefore, we assume that indirect contacts involve only two-hop relaying in the following discussions. We have the DCCP of User *i* with Category *c* with Delay Budget *δ*:

(1) $$Pr_{i}^{c}\left(\delta \right)=1-\left(1-P_{i}^{c}\left(\delta \right)\right)\left(1-{\bar{P}}_{i}^{c}\left(\delta \right)\right)$$

### 3.2. Reward

The reward of a query depends on two factors. First, if a user has a higher likelihood to meet data providers within the remaining delay budget, he or she has a higher possibility to get payed from the query issuer. Second, a query with more copies tends to have a higher value since a user may deliver the query to more data providers in order to obtain more replies. The second factor does not change as long as a query is generated, while the first one is user-dependent. Let $R_{i}^{q}\left(c,\delta \right)$ denote the reward if User *i* trades Query *q* in Category *c* with Delay Budget *δ*. We define $R_{i}^{q}\left(c,\delta \right)$ as:

(2) $$R_{i}^{q}\left(c,\delta \right)=\lambda_{q}\times Pr_{i}^{c}\left(\delta \right)$$

where $\lambda_{q}$ is the appraisal of Query *q* and $Pr_{i}^{c}\left(\delta \right)$ is the DCCP of User *i* in Category *c* with Delay Budget *δ*.

If a query is replied to by a data provider, User *i* obtains the reply. Let ${\hat{R}}_{i}^{\hat{q}}\left(c,\delta \right)$ denote the reward if User *i* trades Reply $\hat{q}$ in Category *c* with Delay Budget *δ*. We define ${\hat{R}}_{i}^{\hat{q}}\left(c,\delta \right)$ as:

(3) $${\hat{R}}_{i}^{\hat{q}}\left(c,\delta \right)=\lambda_{\hat{q}}\times {\hat{Pr}}_{i}^{j}\left(\delta \right)$$

where $\lambda_{\hat{q}}$ is the appraisal of Reply $\hat{q}$ and ${\hat{Pr}}_{i}^{j}\left(\delta \right)$ is the delay-constrained reply contact probability (DRCP), i.e., the probability that User *i* meets Query Issuer *j* directly or indirectly within Delay Budget *δ*. The calculation of DRCP is the same as DCCP, but based on individual users instead of categories.

The reward of a feedback depends on two factors. First, a user only gains credit when he or she delivers the feedback to the data provider; therefore, the reward depends on the contact probability with the data provider. Second, the data provider only pays for the first copy of the feedback. The more the copies, the lower the probability for a copy to be delivered to the data provider before other copies. Let ${\overset{ˇ}{R}}_{i}^{f}\left(c,\delta \right)$ denote the reward if User *i* trades Feedback *f* in Category *c* within Delay Budget *δ*. We define ${\overset{ˇ}{R}}_{i}^{f}\left(c,\delta \right)$ as:

(4) $${\overset{ˇ}{R}}_{i}^{f}\left(c,\delta \right)=\frac{\lambda_{f}\times {\overset{ˇ}{Pr}}_{i}^{k}\left(\delta \right)}{\gamma_{f}}$$

where $\lambda_{f}$ is the appraisal of Feedback *f* and ${\overset{ˇ}{Pr}}_{i}^{k}\left(\delta \right)$ is the delay-constrained feedback contact probability (DFCP), i.e., the probability that User *i* meets the Data Provider *k* directly or indirectly within Delay Budget *δ*. The calculation to obtain DFCP is similar to DRCP. $\gamma_{f}$ is the deliveries of Feedback *f*.

### 3.3. Utility Function

When User *i* meets another user, he or she needs to decide whether or not to exchange queries, replies and feedbacks with the latter. Due to his or her selfish nature, User *i* wants to maximize his or her own reward if the data packet exchange happen. The utility function used by User *i* to trade the queries, replies and feedbacks is given as follows:

(5) $$\begin{array}{cc} U_{i} & =\sum_{q\in \hat{\phi }\left(i\right)}R_{i}^{q}\left(c,\delta \right)-\sum_{q\in \phi (i)}R_{i}^{q}\left(c,\delta \right)\;\\ & +\sum_{\hat{q}\in \hat{\varphi }\left(i\right)}{\hat{R}}_{i}^{\hat{q}}\left(c,\delta \right)-\sum_{\hat{q}\in \varphi \left(i\right)}{\hat{R}}_{i}^{\hat{q}}\left(c,\delta \right)\;\\ & +\sum_{f\in \hat{\psi }\left(i\right)}{\overset{ˇ}{R}}_{i}^{f}\left(c,\delta \right)-\sum_{f\in \psi (i)}{\overset{ˇ}{R}}_{i}^{f}\left(c,\delta \right) \end{array}$$

where $U_{i}$ is the utility function of User *i*, ϕ(i) and $\hat{\phi }\left(i\right)$, φ(i) and $\hat{\varphi }\left(i\right)$, and ψ(i) and $\hat{\psi }\left(i\right)$ are the set of queries, replies and feedbacks in Category *c* before and after the exchange, respectively.

### 3.4. Overview of the Proposed Scheme

To facilitate our discussion, we assume that each data packet is associated with a category, a sequence number, an appraisal and deliveries. Let $L_{i}$, ${\hat{L}}_{i}$, ${\overset{ˇ}{L}}_{i}$ denote the set of queries, replies and feedbacks at User *i*, respectively.

1. When User *i* meets User *j*, he or she first updates his or her DCCP, DRCP and DFCP, respectively. User *i* creates his or her candidate query, reply and feedback lists, i.e., $L_{i}=L_{j}-\left(L_{i}\cap L_{j}\right)$, ${\hat{L}}_{i}={\hat{L}}_{j}-\left({\hat{L}}_{i}\cap {\hat{L}}_{j}\right)$ and ${\overset{ˇ}{L}}_{i}={\overset{ˇ}{L}}_{j}-\left({\overset{ˇ}{L}}_{i}\cap {\overset{ˇ}{L}}_{j}\right)$. $L_{i}$, ${\hat{L}}_{i}$ and ${\overset{ˇ}{L}}_{i}$ are sorted in a decreasing order of the reward of data packets. Note that they are the queries, replies and feedbacks at User *j*, but not at User *i*.
2. User *i* checks if he or she is a data provider for any query in $L_{i}$. If he or she is, User *i* requests those queries from User *j*. For each received query, e.g., Query *q*, User *i* replies to it, gives it back and authorizes User *j* to deliver it to the query issuer. Upon receiving the reply, User *j* decreases the deliveries of Query *q* by one, i.e., $\gamma_{q}=\gamma_{q}-1$.
3. User *i* checks if it is a query issuer of any reply in ${\hat{L}}_{i}$. If it is, User *i* pays User *j* a number of credits, which are equal to the appraisal of the reply, and User *j* removes the reply from ${\hat{L}}_{j}$.
4. User *i* checks if he or she is a receiver for any feedback in ${\overset{ˇ}{L}}_{i}$. If he or she is, User *i* pays User *j* a number of credits, which are equal to the appraisal of the feedback, and User *j* removes the feedback from ${\overset{ˇ}{L}}_{j}$. At the same time, User *j* examines if he or she is a data provider for queries, a query issuer for replies or a receiver for feedbacks similarly.
5. Users *i* and *j* bargain about which queries, replies and feedbacks should be traded. The bargaining process is formulated as a two-person cooperative game, and the Nash theorem is applied to reach the optimal solution. Users *i* and *j* exchange queries, replies and feedbacks pair by pair according to the Nash bargaining solution.

We summarize the description in Algorithm 1.

**Algorithm 1:** Incentive algorithm for delay-constrained data query and feedback.
1:When User *i* meets User *j*, User *i* updates his or her DCCP, DRCP and DFCP and creates his or her candidate query, reply and feedback lists, i.e., $L_{i}=L_{j}-\left(L_{i}\cap L_{j}\right)$, ${\hat{L}}_{i}={\hat{L}}_{j}-\left({\hat{L}}_{i}\cap {\hat{L}}_{j}\right)$ and ${\overset{ˇ}{L}}_{i}={\overset{ˇ}{L}}_{j}-\left({\overset{ˇ}{L}}_{i}\cap {\overset{ˇ}{L}}_{j}\right)$.2:**if** User *i* is the data provider for query *q* in $L_{i}$ **then**3: replies to the query and gives back to User *j*;4: $L_{i}=L_{i}-\left\{q\right\}$;5: $\gamma_{q}=\gamma_{q}-1$;6:**else if** User *i* is the query issuer for reply $\hat{q}$ in ${\hat{L}}_{i}$ **then**7: pays a number of credits to User *j*;8:**else if** User *i* is the receiver for feedback *f* in ${\overset{ˇ}{L}}_{i}$ **then**9: pays a number of credits to User *j*;10:
**end if**
11:Users *i* and *j* exchange queries, replies and feedbacks pair by pair according to the Nash bargaining solution.

### 3.5. Game Theory Model

The two-person cooperative game allows players to reach a binding agreement based on the conflicting interests. Given the selfish nature of the two persons, the binding agreement, once reached, must promote the interest of both persons.

The solution for a two-person cooperative game is given by:

(6) $$\left({\hat{U}}_{i},{\hat{U}}_{j}\right)=arg\;\max_{(U_{i},U_{j})\in S}\left(U_{i}-D_{i}\right)\times \left(U_{j}-D_{j}\right)$$

where $\left({\hat{U}}_{i},{\hat{U}}_{j}\right)$ is the optimal solution, also called the Nash solution; ${\hat{U}}_{i}$ and ${\hat{U}}_{j}$ are the utility gain of User *i* and User *j* in the Nash solution, respectively; $U_{i}$ and $U_{j}$ are the utility gain of User *i* and User *j*, respectively; ($U_{i}$, $U_{j}$) forms the utility gain space *S*; ($D_{i}$, $D_{j}$) is the status quo point in space *S*, usually defined as the utility gain of no cooperation, i.e., (0,0); $\left(U_{i}-D_{i}\right)\times \left(U_{j}-D_{j}\right)$ is called the Nash product. What the Nash theorem [36] says is that, for a two-person cooperative game, the solution ($U_{i}$, $U_{j}$) that maximizes the Nash product $\left(U_{i}-D_{i}\right)\times \left(U_{j}-D_{j}\right)$ is optimal.

While the optimal solution can be obtained by applying the two-person cooperative game, it is non-trivial to get such an optimal solution. Since queries, replies and feedbacks are traded separately, we take query trading as an example. We assume the final traded query lists are $L_{i}^{\prime }$ and $L_{j}^{\prime }$. We define $l=∥L_{i}^{\prime }∥=∥L_{j}^{\prime }∥$, $m=∥L_{i}∥$ and $n=∥L_{j}∥$. Apparently, l≤m and l≤n. We assume m≤n. In order to obtain the optimal solution, the total number of query exchanges should be $\sum_{l=0}^{m}\left(C_{m}^{l}\times C_{n}^{l}\right)=O\left(2^{m}\right)$. We can see that the computational cost to get the optimal solution is exceedingly high due to the great number of data packets in the list, rendering it un-scalable to the real implementation. Therefore, we propose a heuristic algorithm by trading only one pair of data packets at a time. The algorithm selects the data packet pair that results in the maximum Nash product for trade. Therefore, the computational complexity is O(mn). The selection process repeats until $L_{i}^{\prime }$ or $L_{j}^{\prime }$ is empty, or there no feasible solution for Equation (6).

## 4. Distributed Online Auction Algorithm

Since replies and feedbacks are not real credits and cannot be used to pay for delivery service directly, even if the trading process helps cashing them in more quickly, there is a possibility that some users may starve due to being out of real credits, and some users may be wealthy, withholding enough credits. If those two users meet and the wealthy user can buy some replies and/or feedbacks from the starving one using real credits, the credit circulation in the network will be greatly improved. Furthermore, this process also does good to both. From the perspective of the starving user, he or she is desperate to get real credits to initiate or pay for his or her own query dissemination process, and he or she is happy to sell some replies and/or feedbacks, even if less than the reward. From the perspective of the wealthy user, the reason why he or she is willing to buy the data packets is that he or she may get profits from cashing in replies from query issuers and feedbacks from data providers. In this way, both users benefit from the data packet buying/selling process. We formulate this process as an auction model.

Now, we come to the question of how the seller decides which bid he or she should accept or reject. Since the seller meets the buyers in a subsequent order and he or she does not meet Buyer *A*, Buyer 1 places two credits for a reply, and he or she accepts the bid and sell the reply for two credits. While at time $t_{2}$, Buyer *B* may place five credits for the same reply. In this case, the seller does not find the best buyer for the reply and loses the chance to maximize his or her gain. Thus, it is essential to choose the best buyer to make an irrevocable decision to sell the reply. In general, User *i* may meet a sequence of users, similar to a stochastic process. He or she must make an adaptive, online decision on which bid should be accepted, in order to achieve the optimal gain. We observe that the online auction process matches the optimal stopping problem with a finite horizon and tries to find the best buyer for the replies and/or feedbacks among all of the buyers.

Based on the above observation, we propose a distributed online algorithm based on the optimal stopping theory. We first present an analysis followed by the protocol design.

### 4.1. Analysis

Since we are concerned about the problem of accepting a bid within a certain delay budget, we propose a distributed approach based on the stopping rule problem with a finite horizon. A stopping rule problem has a finite horizon if there is a known upper bound on the number of stages at which one may stop.

In order to facilitate the following discussion, we denote the remaining delay budget *δ* as T-τ, then we denote the expected reward of a reply with remaining delay budget (T-τ) as $X_{\left(T-\tau \right)}$, and we define $Y_{\tau }$ as the return when User *i* decides to accept a bid after *τ* time slots, i.e.,

(7) $$Y_{\tau }=X_{\left(T-\tau \right)}=X_{T}\cdot g_{\tau }$$

where (T-τ) is the remaining delay budget to deliver the reply and $g_{\tau }$ means that the further delivery is at the cost of the decrease in the delay budget. We denote the pdf of *X* as f(x).

For the online auction problem, User *i* will meet a certain user (or users), which may help he or she to deliver the reply at time slot *τ*, with $Y_{\tau }$ observed. User *i* may decide to stop at time slot *τ* or to continue to meet other users. Therefore, the online auction problem can be considered as an optimal stopping problem with an objective to find the optimal stopping time that maximizes the expected return, i.e.,

(8) $$\tau^{*}≜\arg\max_{\tau }\left[E\left(Y_{\tau }\right)\right],Y^{*}≜\underset{\tau }{\sup}\left[E\left(Y_{\tau }\right)\right]$$

We define $V_{\tau }$ as the maximum return the user can obtain if the user accepts a bid with Delay Budget *T* after *τ* time slots. At *τ*, we compare the return for stopping, namely $Y_{\tau }$, with the return we expect to be able to get by continuing and using the optimal rule for time slots τ+1 through *T*, which at time slot *τ* is $E(V_{\tau +1}\left(Y_{\tau +1}\right))$, i.e.,

(9) $$V_{\tau }=\max\left\{Y_{\tau },E\left(V_{\tau +1}\left(Y_{\tau +1}\right)\right)\right\}$$

From Equation (9), we can see that $E(V_{\tau +1}\left(Y_{\tau +1}\right))$ serves as a threshold in the sense that if $Y_{\tau }$ is above the threshold, it is optimal for the user to accept the bid. We define the threshold at time slot *τ* as:

(10) $$\rho_{\tau }^{*}=E\left(V_{\tau +1}\left(Y_{\tau +1}\right)\right)$$

Then, we can obtain the optimal stopping strategy of the online auction problem as follows.

**Theorem 1.**  *For the online auction problem, it is optimal for the user to accept the bid if the following condition is satisfied at τ,*

(11) $$\tau^{*}=\underset{\tau }{\inf}\left\{\tau >0:Y_{\tau }\geq \rho_{\tau }\right\}$$

**Theorem 2.**  *For the online auction problem, the threshold of the optimal stopping strategy is given by:*

(12) $$\rho_{T}^{*}=0$$

(13) $$\rho_{T-1}^{*}=g_{T}\int_{X_{d}}^{X_{max}}xf\left(x\right)dx$$

⋮

(14) $$\rho_{\tau^{*}}^{*}=g_{\tau +1}\int_{\frac{\rho_{\tau +1}^{*}}{g_{\tau +1}}}^{X_{max}}xf\left(x\right)dx+\rho_{\tau +1}^{*}\int_{X_{d}}^{\frac{\rho_{\tau +1}^{*}}{g_{\tau +1}}}f\left(x\right)dx$$

**Proof.**  When *T* time slots are consumed, the reply cannot be delivered to the query issuer due to the expiration of the reply; therefore, the reward is zero, i.e., $\rho_{T}^{*}=0$. Then, we have $Y_{T}\geq \rho_{T}^{*}$. Then, according to Equation (10), we can obtain $\rho_{T-1}^{*}$ as:

(15) $$\begin{array}{cc} \rho_{T-1}^{*}= & E\left[V_{T}\right]=E\left[Y_{T}\right],\;\\ = & g_{T}E\left[X_{T}\right]=g_{T}\int_{X_{d}}^{X_{max}}xf\left(x\right)dx \end{array}$$

☐

Combining Equations (12) and (13), we can next compute ${\left\{\rho_{\tau }^{*}\right\}}_{\tau =0}^{T-2}$ by the backward induction as:

(16) $$\begin{array}{c} \rho_{\tau }^{*}=E\left[\max\left\{Y_{\tau },\rho_{\tau +1}^{*}\right\}\right]=E\left[\max\left\{g_{\tau }X_{\tau },\rho_{\tau +1}^{*}\right\}\right]=\\ g_{\tau +1}\int_{\frac{\rho_{\tau +1}^{*}}{g_{\tau +1}}}^{X_{max}}xf\left(x\right)dx+\rho_{\tau +1}^{*}\int_{X_{d}}^{\frac{\rho_{\tau +1}^{*}}{g_{\tau +1}}}f\left(x\right)dx \end{array}$$

### 4.2. Protocol Design

After the query is replied to by the data provider, it will meet a set of intermediate users. As introduced in Section 3.2, let ${\hat{R}}_{i}^{\hat{q}}\left(c,\tau \right)$ denote the expected reward of Reply $\hat{q}$ in Category *c* at User *i* that intends to get payed from the query issuer.

We consider that User *i* meets User *j* at time slot *τ*; User *j* places a bid $b_{j}$; if the reply is not sold to User *j*, then in the next time slot τ+1, the expected reward of User *i* to deliver the reply will become ${\hat{R}}_{i}^{\hat{q}}\left(c,\tau -1\right)$.

According to Theorem 1, User *i* accepts the bid if and only if the following condition is satisfied:

(17) $$b_{j}\geq {\hat{R}}_{i}^{\hat{q}}\left(c,\tau -1\right)$$

Note that feedbacks can be handled similarly. Since the communication opportunity is low, transmission is often between two users only. If more than two users are within communication range, we assume an underlying medium access control protocol (e.g., IEEE 802.11) that randomly selects one user as the seller and another as the buyer.

## 5. Performance Evaluation

We have carried out simulations to demonstrate the efficiency of the proposed schemes. In this section, we first introduce our simulation setup and then present simulation results.

### 5.1. Simulation Setup

We have compared the performance of different schemes as summarized in Table 2 and Table 3: the “selfish” scheme, where no cooperation exists among users, and thus, queries are replied to and feedbacks delivered only when query issuers meet data providers directly; the “cooperative” scheme, where users are fully cooperative and always choose the most valuable data packets to carry; the “TFT” scheme, where a user forwards as much traffic for a neighbor as the neighbor forwards for him or her [37]; and our proposed incentive schemes denoted by “incentive” and “incentive with auction”.

We have evaluated our proposed schemes under two real-world traces, i.e., the Cambridge Haggle trace and the UMassDieselNettrace. The former involves 98 iMotes and Bluetooth devices and runs for a total period of about three days. The latter is based on a MOSNtestbed, which is constructed by 37 transit buses, serving an area of approximately 150 square miles for a period of about two weeks in 2006.

We assume there are 30 categories in the network. The queue size of each user is 250. The initial credit for each user is 120. The query issuer generates one query every 15 min in a random category with the delay budget randomly distributed from one hour to 10 h. For the online auction process, the bid price is set to be randomly distributed from 70% to 100% of the reward of the query or feedback when it is generated. The claimed expertise in each category for each user is randomly set and learned and updated during the simulation.

### 5.2. Performance Comparison

We are interested in the following metrics for performance evaluation: query reply rate, query delay and transmission overhead. The query reply rate is defined as the ratio of the total number of replied queries to the total number of queries generated. Query delay is a measure of how long a query issuer waits to get a query reply. Transmission overhead is defined as the ratio of the total number of transmissions to the total number of replied queries.

Table 2 and Table 3 compare the overall performance of different schemes based on the Haggle trace and the DieselNet trace, respectively. The high query reply rate of “incentive” is attributed to the fact that users are well stimulated by employing the reward of data packets, leading to highly efficient data transmission. It seems counter-intuitive that the query reply rate of “cooperative” is lower than that of “incentive”, because users are all altruistic with respect to helping each other by always choosing the most valuable data packets to carry. It actually makes sense that cooperation always makes data packets aggregate quickly and, thus, dropped due to queue overflow. While “TFT” considers selfishness, its query reply rate is lower than “incentive”, because maintaining a mutual forwarding balance wastes useful contact opportunities. Moreover, we can see that “Incentive with auction” further improves the query reply rate. This is because the auction process makes users have more chances to cash in the replies and feedbacks and to get credits to pay for their delivery service and initiate more query dissemination and feedback retrieval. Finally, users under “selfish” do not cooperate at all, resulting in the lowest query reply rate.

The shorter delays of “cooperative”, “incentive” and “incentive with auction” are contributed to by the fact that they leverage the packet value to estimate the probability to deliver the packet and choose the best routes to forward it. “Selfish” exhibits the longest delay because a query is delivered and replied to only when the query issuer and the data provider meet directly. Although the source in the “TFT” scheme specifies the complete route for each generated packet, packets may not always follow the best routes due to the “TFT” constraint and results in a longer delay. Moreover, “cooperative” has a much higher overhead than “incentive”, because its altruism leads to more packets to be duplicated and distributed in the network. In contrast, the proposed “incentive” and “incentive with auction” achieve very low overhead, because a user receives a query only if the query is redeemed as a benefit for the user. Clearly, the overhead of “selfish” is always one, because a user only replies to the queries in its own categories.

**Figure 2:**
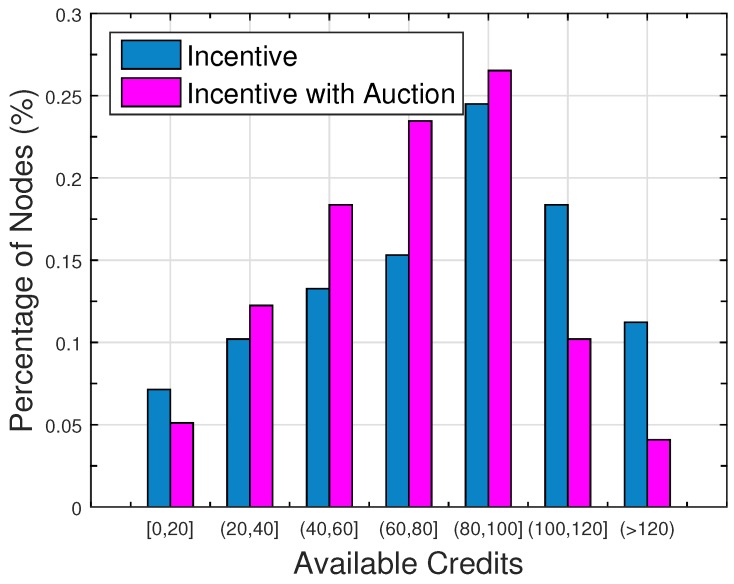
Distribution of available credits.

Figure 2 illustrates the distribution of available credits under “incentive” and “incentive with auction”. Credits are consumed for replies and feedbacks. The more credits a user owns, the more queries it can disseminate and the more feedbacks it can retrieve. We can see that most users have available credits less than 120; this is because they all hold some replies and feedbacks waiting to be delivered to the query issuers and data providers. Fifty four percent of users keep their available credits around 40 to 100 in “incentive”compared to 68% in “incentive with auction”. This indicates that the auction process helps users keep a better balance of credit retrieval and consuming.

**Figure 3:**
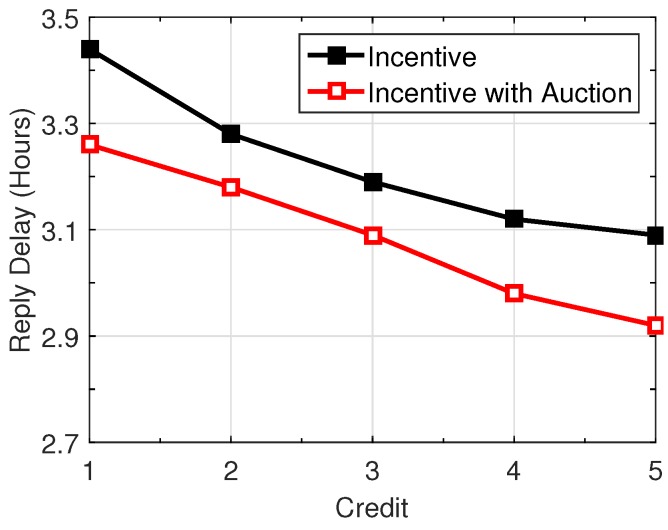
Impact of credit amount on reply delay.

Figure 3 shows the performance of the query reply delay if we change the credit amount of one query. A user is chosen as an example, while similar results are observed for other users, as well. We increase its credit amount from 1 to 6, while all other queries keep one. As can be seen, the query reply delay decreases with the increase of credit. This is because a higher credit indicates more rewards if a user successfully delivers the query, resulting in stronger incentives to stimulate nodal collaboration.

**Figure 4:**
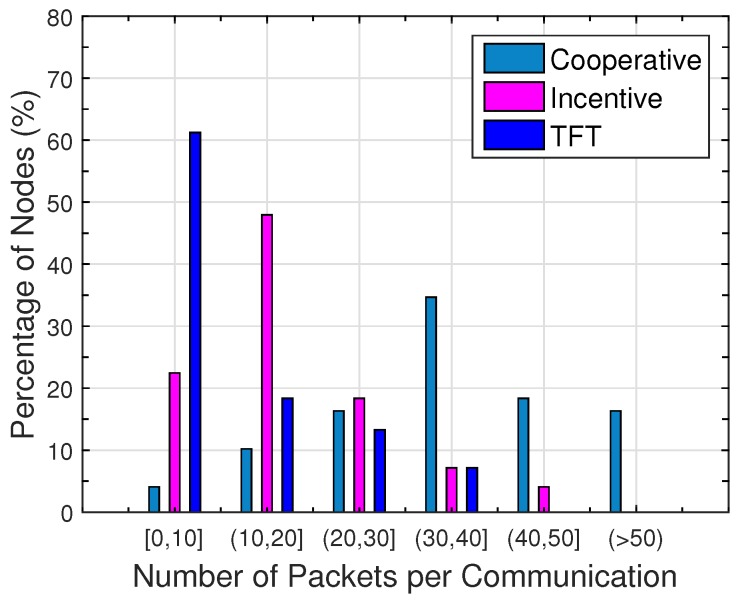
Distribution of the packet exchange.

Figure 4 illustrates the average number of packets exchanged when two users meet. We can see that “incentive” exchanges more packets than “TFT”, but less than “cooperative”. The more packets are exchanged, the more resources are consumed. Sixty-one-point-two percent of users in “TFT” exchange less than 10 packets per communication, because the constraints in “TFT” enforce bilateral balances. Sixty-nine-point-four percent of users in “cooperative” exchange more than 30 packets per communication. In “incentive”, users keep a good balance between their own gains and contributions to the network. Forty-eight percent of users exchange 10 to 20 packets per communication. Since “incentive with auction” does not impact the packet exchange factor, we omit its performance here.

**Figure 5:**
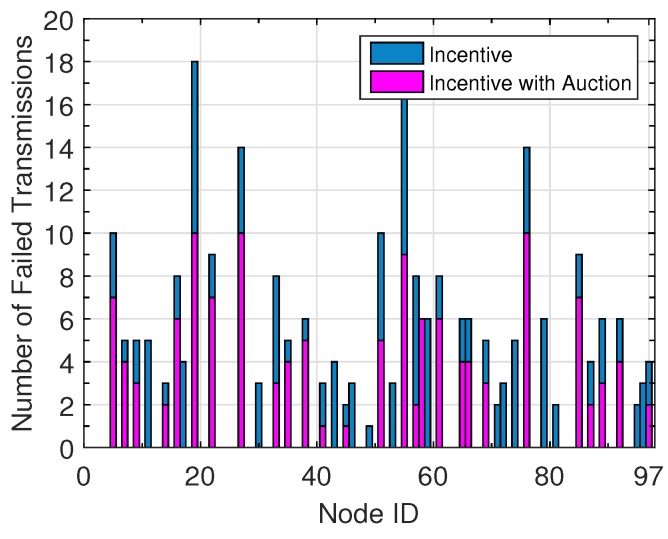
Distribution of the failed transmissions.

The distribution of failed transmissions among users is depicted in Figure 5. The failed transmissions are due to the lack of cooperation opportunities, which consequently leads to credit shortage. We can see that 60% of the users do not have any failed transmissions, and the average number of failed transmission is less than five, showing that the two-person cooperative game helps both users obtain gains and prevents unilateral benefit. Furthermore, the auction process helps about 27.6% of users reduce their failed transmissions, thus improving the credit circulation in the network.

**Figure 6:**
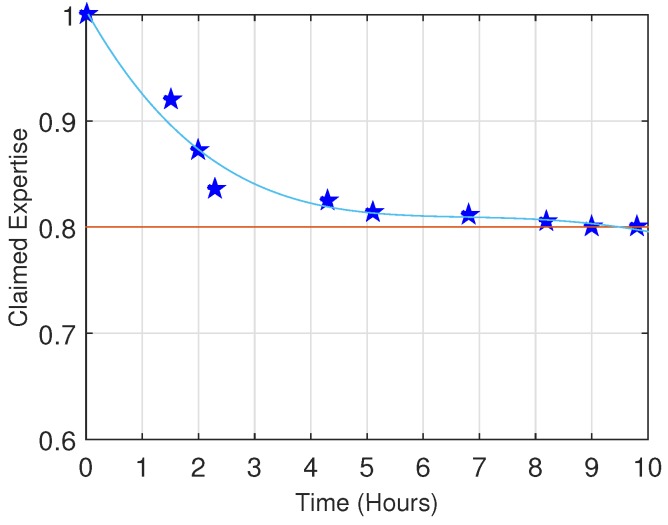
Convergence of expertise.

Figure 6 shows the convergence of the claimed expertise to the ground truth. We randomly choose a user as an example. As we can see, the feedback mechanism effectively adjusts the user’s expertise, gradually approaching the true value within a few hours.

**Figure 7:**
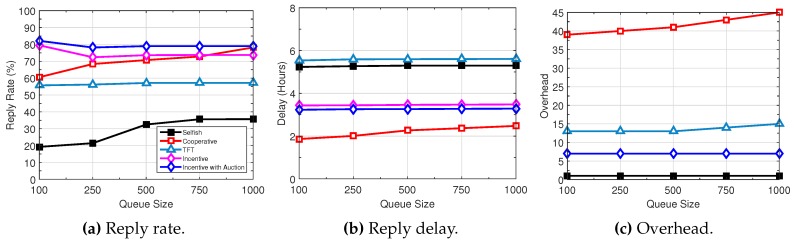
Performance trend with increasing queue size.

**Figure 8:**
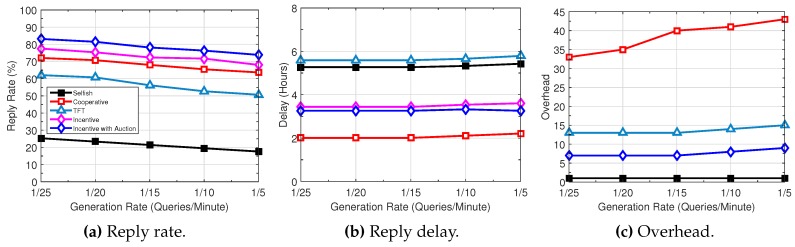
Performance trend with increasing generation rate.

**Figure 9:**
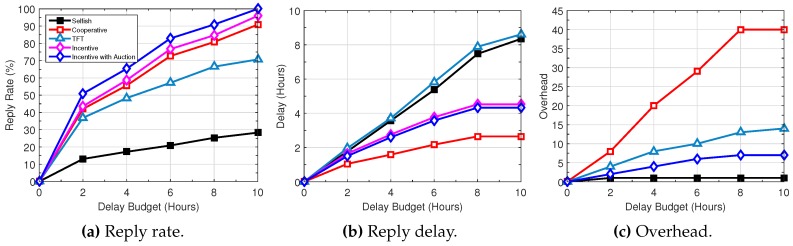
Performance trend with increasing delay budget.

Figure 7, Figure 8 and Figure 9 illustrate the performance trend with the variation of several network parameters based on the Haggle trace. The DieselNet trace shows a similar trend. With the increase of queue size, the query reply rate of all schemes increases (see Figure 7). Particularly, “cooperative” increases significantly, because a longer queue allows more data packets to be buffered for a longer time, thus increasing the probability of query delivery. At the same time, query delay decreases. On the other hand, the overhead increases rapidly. Since “TFT” is constrained by the amount of traffic forwarded for others and “incentive” and “incentive with auction” exchange data packets based on self interests and aim to maximize their rewards, the increase of queue size has marginal impact on the performance of “TFT” and the proposed “incentive” and “incentive with auction”.

The impact of traffic load is illustrated in Figure 8; we vary the packet generation rate. With the increase of the query generation rate, the query reply rate decreases. Given the limited resources at individual users, the higher the generation rate, the longer the queries and replies need to reside at the sources and intermediate users, leading to longer reply delay. The overhead increases as well, since more data packets are duplicated during their transmissions.

Figure 9 compares the performance by varying the delay budget of queries. Figure 9a shows that with the increase of the delay budget, all schemes achieve a higher query reply rate. We notice from Figure 9b that most queries can be replied to within eight hours in “cooperative”, “incentive” and “incentive with auction”, while “selfish” and “TFT” need to keep queries longer in the queue. Moreover, the overhead of all schemes increases with the increase of the delay budget. This is because a longer delay budget allows queries to stay longer in the queue, resulting in a better chance to be duplicated and distributed in the network.

## 6. Conclusions

We have proposed an incentive scheme to stimulate cooperation between selfish users for data query and feedback in mobile opportunistic crowdsensing. Queries, replies and feedbacks can be traded between mobile users. We have proposed an effective mechanism to define rewards for queries, replies and feedbacks and formulate user interaction as a two-person cooperative game. To improve the credit circulation, we have considered a bid placing problem. We have proven that the problem can be formulated as an optimal stopping problem, given a closed-form expression for the threshold of the optimal stopping strategy and then developed an online auction algorithm based on the optimal stopping strategy that makes an efficient decision on every bid. Extensive simulations have been carried out based on real-world traces to evaluate the proposed schemes. We leave further study on a testbed experiment using off-the-shelf Nexus tablets to demonstrate the feasibility and efficiency of the proposed algorithms and to gain useful empirical insights, as well as possible consideration of data fusion as our future works.

## Figures and Tables

**Table 1:** The main definitions in the paper.

Definition	Description
$\gamma_{q}$	The deliveries of Query *q*
$\gamma_{f}$	The deliveries of Feedback *f*
$\lambda_{q}$	The appraisal of Query *q*
$\lambda_{f}$	The appraisal of Feedback *f*
$Pr_{i}^{c}\left(\delta \right)$	The delay-constrained category contact probability (DCCP)
$P_{i}^{c}\left(\delta \right)$	The direct delay-constrained contact probability of User *i* with data providers in Category *c* with remaining Delay Budget *δ*
${\bar{P}}_{i}^{c}\left(\delta \right)$	The indirect delay-constrained contact probability of User *i* with data providers in Category *c* with remaining Delay Budget *δ*
$R_{i}^{q}\left(c,\delta \right)$	The reward if User *i* trades Query *q* in Category *c* with Delay Budget *δ*
${\hat{R}}_{i}^{\hat{q}}\left(c,\delta \right)$	The reward if User *i* trades Reply $\hat{q}$ in Category *c* with Delay Budget *δ*
${\hat{Pr}}_{i}^{j}\left(\delta \right)$	The delay-constrained reply contact probability (DRCP)
${\overset{ˇ}{R}}_{i}^{f}\left(c,\delta \right)$	The reward if User *i* trades Feedback *f* in Category *c* within Delay Budget *δ*
${\overset{ˇ}{Pr}}_{i}^{k}\left(\delta \right)$	The delay-constrained feedback contact probability (DFCP)

**Table 2:** Overall performance comparison based on the Haggle trace.

	Reply Rate	Reply Delay	Overhead
Selfish	0.21	5.27 h	1
Cooperative	0.68	2.01 h	40
TFT	0.56	5.59 h	13
Incentive	0.72	3.44 h	7
Incentive with Auction	0.78	3.26 h	7

**Table 3:** Overall performance comparison based on the DieselNettrace.

	Reply Rate	Reply Delay	Overhead
Selfish	0.19	12.19 h	1
Cooperative	0.79	6.14 h	42
TFT	0.70	14.16 h	16
Incentive	0.81	6.98 h	9
Incentive with Auction	0.85	6.44 h	9
